# A new primate model of hypophyseal dysfunction

**DOI:** 10.1038/s41598-021-90209-3

**Published:** 2021-05-24

**Authors:** Teppei Kawabata, Hidetaka Suga, Kazuhito Takeuchi, Yuichi Nagata, Mayu Sakakibara, Kaori Ushida, Chikafumi Ozone, Atsushi Enomoto, Ikuo Kawamoto, Iori Itagaki, Hideaki Tsuchiya, Hiroshi Arima, Toshihiko Wakabayashi

**Affiliations:** 1 Department of Neurosurgery, Graduate School of Medicine, Nagoya University, Nagoya, Japan; 2 Department of Endocrinology and Diabetes, Graduate School of Medicine, Nagoya University, 65 Tsurumai-cho, Showa-ku, Nagoya, 466-8550 Japan; 3 Department of Pathology, Nagoya University Graduate School of Medicine, Nagoya, Japan; 4 Technical Center, Nagoya University, Nagoya, Japan; 5 Department of Endocrinology and Diabetes, Japanese Red Cross Nagoya Daini Hospital, Nagoya, Japan; 6 Research Center for Animal Life Science, Shiga University of Medical Science, Shiga, Japan

**Keywords:** Endocrinology, Medical research

## Abstract

For pituitary regenerative medicine, the creation of a hypophyseal model in monkeys is necessary to conduct future preclinical studies; however, previous studies reported that hypophysectomy in monkeys is not always safe or satisfactory. This study aimed to create a hypophyseal dysfunction model in a cynomolgus monkey using a safer surgical technique and establish the protocol of pituitary hormone replacement therapy for this model. Surgical resection of the pituitary gland of a 7.8-year-old healthy adult cynomolgus male monkey weighing 5.45 kg was performed to create a hypophyseal dysfunction model for future regenerative studies. Endoscopic transoral transsphenoidal surgery was used to perform hypophysectomy under navigation support. These procedures were useful for confirming total removal of the pituitary gland without additional bone removal and preventing complications such as cerebrospinal fluid leakage. Total removal was confirmed by pathological examination and computed tomography. Hypopituitarism was verified with endocrinological examinations including stimulation tests. Postoperatively, the monkey’s general condition of hypopituitarism was treated with hormone replacement therapy, resulting in long-term survival. The success of a minimally invasive and safe surgical method and long-term survival indicate the creation of a hypophyseal dysfunction model in a cynomolgus monkey; hence, this protocol can be employed in the future.

## Introduction

Recently, many studies have examined the differentiation of pluripotent stem cells (iPSCs) into various somatic cells in vitro. Both the transplantation of retinal pigment epithelium derived from human iPSCs and dopamine neurons have been performed in humans. In the evaluation of the safety and effectiveness of the transplantation of the cells from human iPSCs, monkey transplantation experiments were performed before human application ^1,2^.

We developed a technology that differentiates pituitary cells from human iPSCs. We showed that that adrenocorticotropic hormone (ACTH) and corticosterone levels recovered after transplantation in hypophysectomized mice and the survival rate improved ^3,4^. Soon, a technology that differentiates pituitary cells from human iPSCs will be necessary to plan transplantation experiments using a hypophyseal dysfunction model in monkeys ^5^, in which there have been several reports concerning hypophysectomy ^6–9^; however, its mortality rate was 50% with a non-existent protocol for pituitary hormone replacement therapy ^7^. Therefore, it seems inappropriate to create a transplantation model for hypophyseal dysfunction in monkeys based on these previous methods. Endoscopic transsphenoidal surgery is commonly used as a less invasive and effective technique of removing pituitary tumors in humans ^10^. This study aimed to create a hypophyseal dysfunction model in a cynomolgus monkey using a safer surgical technique and establish the protocol of pituitary hormone replacement therapy for this model.

## Methods

### Study approval

The protocol for all experiments was approved by the Ethical Committees of Medical Science of Shiga University (approval number: 2017-3-10) and Nagoya University (approval numbers: 29459, 30105, and 31077). All applicable institutional and/or national guidelines for the care and use of animals were followed. This study was conducted according to the ARRIVE guidelines.

### Animal model

A 7.8-year-old healthy adult male cynomolgus monkey weighing 5.45 kg was used in this study. Regarding reproduction, rhesus monkeys are seasonally reproductive, whereas cynomolgus monkeys reproduce any time of the year. Considering a situation wherein a sufficient number of cases cannot be set, we considered the reproductive cycle of the cynomolgus monkey to be similar to that of humans; so therefore, it was deemed better to consider these monkeys in the experimental model than the rhesus monkey, which has a highly uncertain fertile period. We selected a male cynomolgus monkey because males generally have a larger body than females; hence, we planned to remove the pituitary gland in the monkey for the first time. The monkey was housed in an individual cage and had free access to water. The animal was fed with CLEAR Old-World Monkey Diet (100 g, CMK-2; CLEA Japan, Inc., Tokyo, Japan) and 20 g of sweet potatoes daily. If the monkey developed any intolerable pain, euthanasia was to be performed after an adequate study period as a humane endpoint.

### Preoperative examination

**Figure 1:**
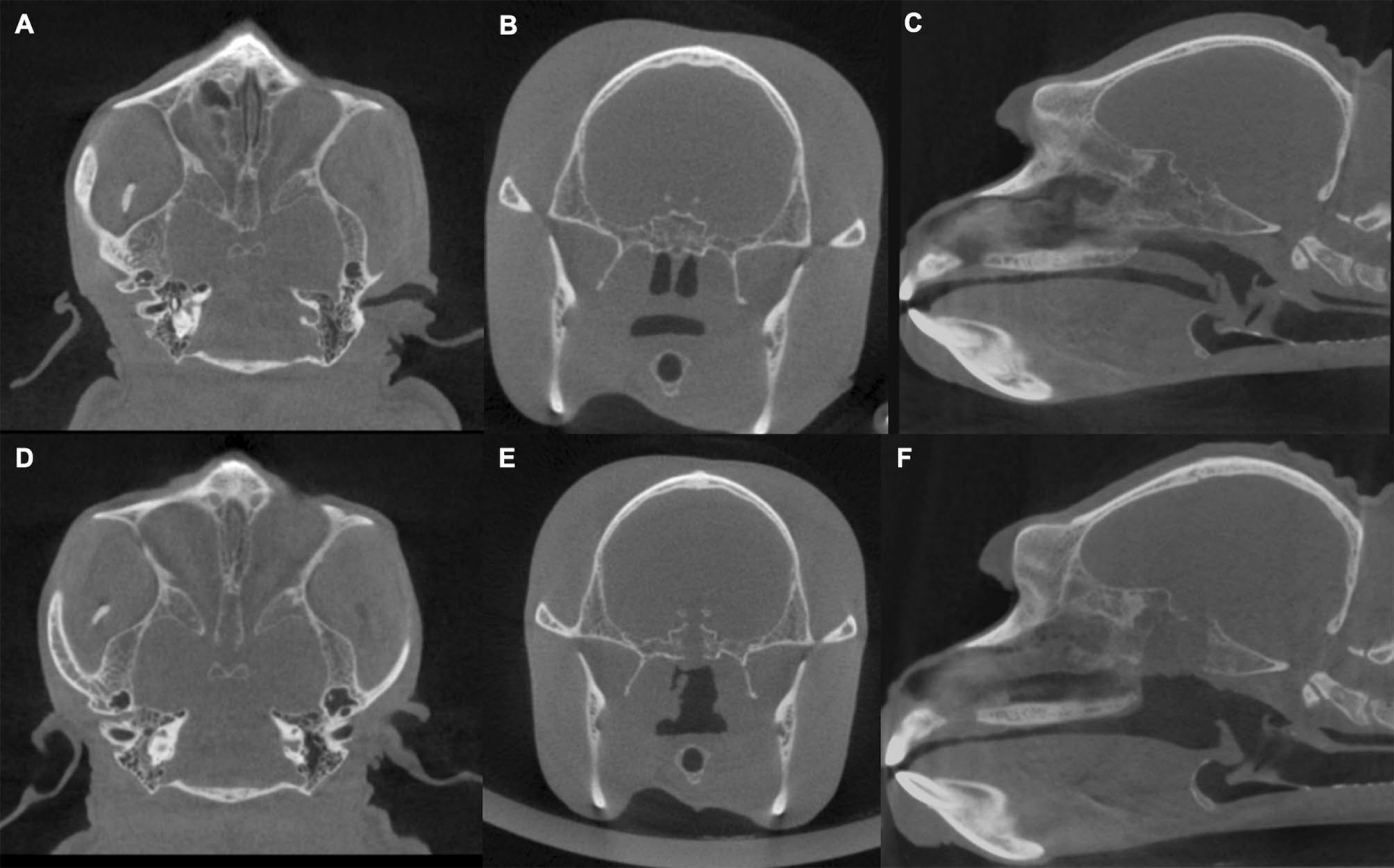
Preoperative and postoperative computed tomography scans. Preoperative axial (**A**), coronal (**B**), and sagittal images (**C**) show the sphenoidal sinus and pituitary fossa in the cynomolgus monkey. Postoperative axial (**D**), coronal (**E**), and sagittal images (**F**) show the bone removal in the sphenoidal sinus.

Blood and computed tomography (CT) examinations for the navigation system were performed preoperatively with the monkey under sedation (Fig. 1A–C). The serum levels of ACTH, cortisol, growth hormone (GH), prolactin, and luteinizing hormone were preoperatively obtained (SRL, Tokyo, Japan) for the evaluation of anterior pituitary function. The thyroid-stimulating hormone could not be accurately measured because of the racial interaction between the human and monkey. Similarly, the arginine vasopressin (AVP) level was measured to assess the posterior pituitary function.

### Anesthesia

Anesthesia was performed, with atropine (50 μg/kg) administered intramuscularly as premedication. Ketamine (5 mg/kg) and xylazine (1 mg/kg) were administered intramuscularly. A tracheal tube was inserted and fixed with the silk blade on the left side of the monkey’s mouth. Both dexamethasone (4 mg/m^2^) and ceftriaxone (20 mg/kg) were administered intravenously just before the surgical procedure as steroid replacement and antibiotic therapies, respectively. Isoflurane was administered by inhalation for anesthesia during the surgical procedure at a concentration of 1–3%.

### Surgical methods

**Figure 2:**
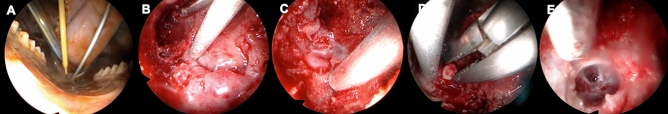
Endoscopic finding during the operation. Soft palate (**A**), drilling of the sphenoid bone (**B**), dura on the sella (**C**), pituitary gland (**D**), and space after the pituitary gland removal (**E**).

The endoscopic transoral transsphenoidal approach was performed for hypophysectomy (Fig. 2A–E). The pituitary gland’s accurate direction was confirmed by a surgical navigation system. The monkey’s head in prone position was slightly rotated toward the operator. The monkey’s mouth was opened as wide as possible by pulling the maxilla’s cuspid upward with the string. The soft palate was linearly incised in the midline; subsequently, the plate was laterally retracted with the silk blade. The mucosa in front of the sphenoid bone was linearly incised in the midline, while the sphenoid bone was exposed. The monkey had a conchal-type sphenoidal sinus; therefore, we drilled the sphenoidal bone using a high-speed drill with a diamond burr. We checked the surgical orientation several times during this procedure with the navigation system, as there were no surgical landmarks inside the conchal-type sphenoid sinus. After removing the sphenoidal bone and the sella floor, we cut the dura’s sella with scissors. We removed the pituitary gland and stalk and froze the histopathological specimen. We confirmed the absence of residual pituitary gland under close inspection through angled endoscopy. The sella was reconstructed with fat grafts harvested from the abdominal subcutaneous fat layer, and fibrin glue was used to prevent postoperative cerebrospinal fluid (CSF) leakage. No hemorrhage or CSF leakage after the reconstruction was confirmed. We sutured the soft palate and completed the operation.

### Postoperative treatment

The intravenous drip infusion was stopped shortly after the monkey regained consciousness. The monkey could eat food after waking up and had free water access. Ceftriaxone (20 mg/kg) was administered intramuscularly for 3 days postoperatively. We considered the intramuscular administration of buprenorphine (4 μg/kg) when the monkey developed pain postoperatively.

**Figure 3:**
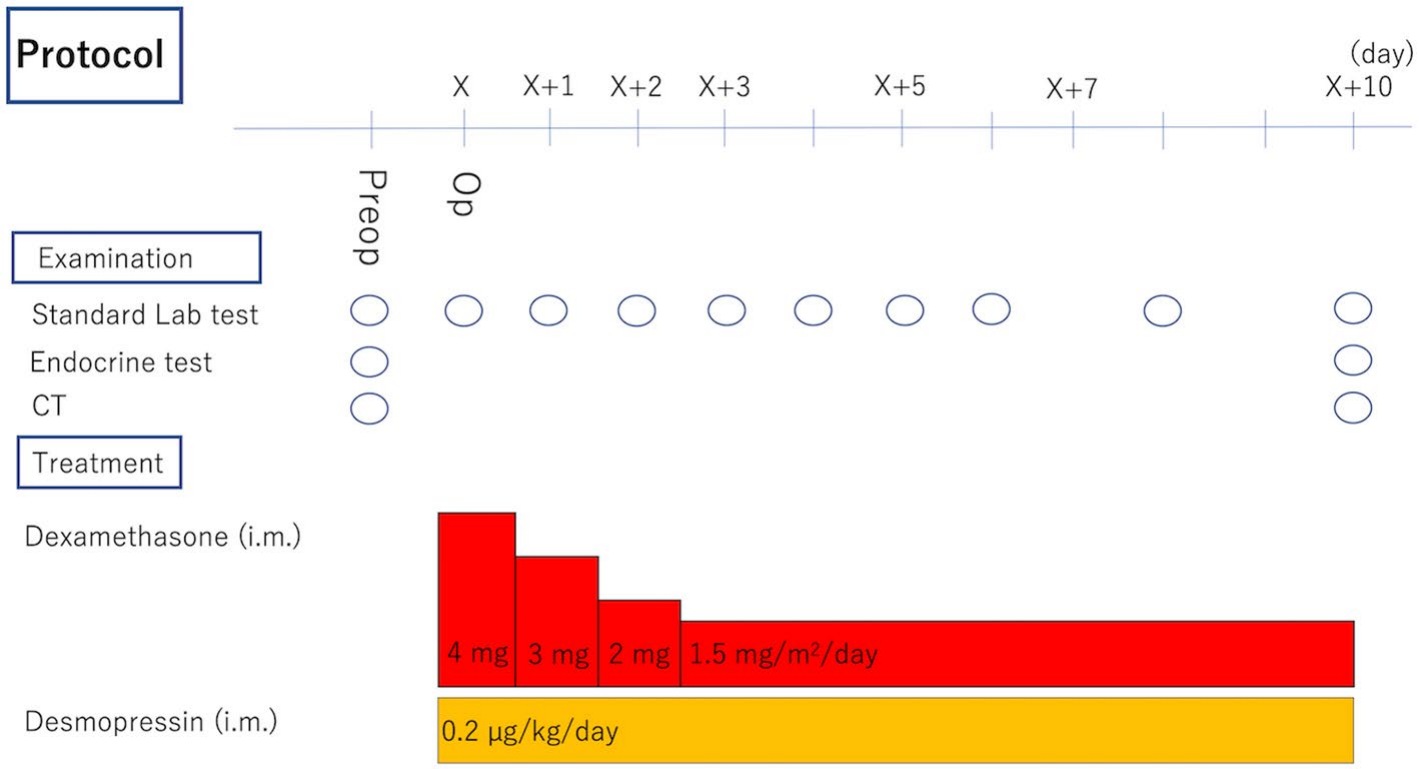
Protocol of perioperative management. *Preop* preoperatively, *Op* operatively, *Lab* laboratory, *i.m.* intramuscularly.

Hypopituitarism was treated with daily hormone replacement, which was administered intramuscularly. The doses of dexamethasone, thyroxin, and vasopressin were determined based on the clinical dose for human hypopituitarism. Dexamethasone was decreased gradually from 4 to 1.5 mg/m^2^/day (Fig. 3), and thyroxin (2 µg/kg/day) was administered orally from 23 days after the operation. Postoperative diabetes insipidus (DI) was treated with vasopressin (0.2 µg/kg/day) and free access to water. The monkey’s water balance was assessed by its body weight and serum sodium (Na) level because it was difficult to accurately measure water intake and urine output.

### Postoperative evaluation

Water and electrolyte imbalance were assessed by blood examinations. Infection was evaluated based on surgical wound, body temperature, and amount of food intake. Hormone stimulation tests and CT were performed with the monkey under sedation at 10 postoperative days (Fig. 1D–F). Anterior pituitary function was measured after the corticotropin-releasing hormone (CRH) (1.5 µg/kg) and growth-hormone-releasing factor (GRF) (1 µg/kg) were intravenously administered. Blood sampling was performed 30 min after the injection, and posterior pituitary function was evaluated based on the presence of DI and blood AVP level. Similarly, total removal of the pituitary gland was also confirmed by histopathological examination.

### Immunohistochemistry

**Figure 4:**
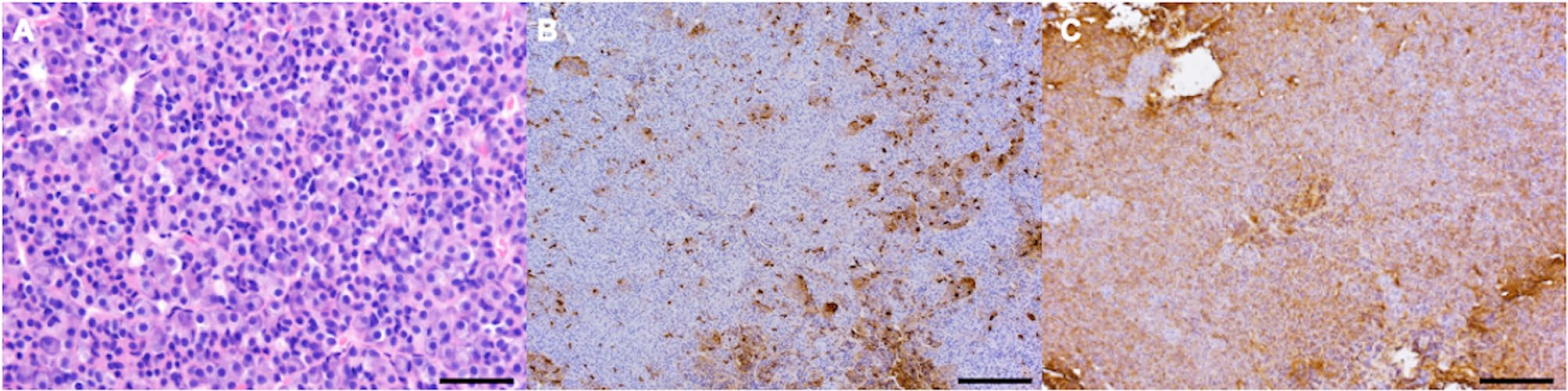
Histopathological examination. Hematoxylin and eosin staining showing the resected anterior pituitary gland (bar = 50 µm). (**A**) The tissue is detected as the immunopositive area using anti-adrenocorticotropic hormone staining (**B**) and anti-growth hormone (**C**) staining (bar = 200 µm).

Excised tissue was fixed in a 10% formalin neutral buffer solution. Paraffin-embedded sections were stained with hematoxylin and eosin. Regarding immunohistochemical staining, deparaffinized sections were blocked with Protein Block (X0909; Agilent Technologies, Santa Clara, CA, USA). Subsequently, sections were incubated with a primary antibody against human ACTH (M3501, Agilent Technologies) or GH (A0570, Agilent Technologies) (Fig. 4A–C). After applying 0.3% hydrogen peroxide in methanol treatment, sections were incubated with the EnVision + System-HRP Labeled Polymer (K4001, K4003; Agilent Technologies). DAB reactions were visualized using the Liquid DAB + Substrate Chromogen system (K3468; Agilent Technologies); counterstaining was performed with hematoxylin.

## Results

Basal state hormone levels were evaluated at 5 days before surgery; results showed 68.9 pg/mL ACTH levels (normal range, 7–63 pg/mL), 25.8 μg/dL cortisol levels (7.07–19.6 μg/dL), 12.8 ng/mL growth hormone (GH) levels, and 0.6 pg/mL AVP levels (Table 1). The surgical procedure was successful without complications. The postoperative course, based on blood test results, is shown in Table 2. CRH and GRF stimulation tests were performed on postoperative day 10, revealing pituitary function response (Table 1). The cortisol level was 0.51 μg/dL, and ACTH, AVP, and GH levels were undetectable (Table 1). Postoperative CT showed minimal bone defects on the pituitary gland site. Pathological evaluation showed an immuno-positive area on ACTH and GH resected tissue.

**Table 1:** Preoperative and postoperative hormone levels.

	Preoperatively	10 days postoperatively	2 years postoperatively
ACTH level (pg/mL)	68.9	Undetectable	Undetectable
Cortisol level (μg/dL)	25.8	0.51	0.44
GH level (ng/mL)	12.8	Undetectable	Undetectable
AVP level (pg/mL)	0.6	Undetectable	Undetectable

Preoperative hormone levels were measured in the basal state. Postoperative anterior pituitary function was measured after stimulation with the intravenous administration of corticotropin-releasing hormone and growth-hormone releasing factor. The preoperative and postoperative day 10 AVP levels were measured in the basal state. At 2 years postoperatively, the AVP level was measured after water deprivation (2 h for computed tomography imaging).

*ACTH* adrenocorticotropic hormone, *GH* growth hormone, *AVP* arginine vasopressin.

**Table 2:** Time course of postoperative blood examination in the monkey postoperatively.

	Na level (mEq/L)	K level (mEq/L)	Cl level (mEq/L)	BUN level (mg/dL)	Cre level (mg/dL)	Hct count (%)	Hb level (g/dL)
POD 0	153	3.7	115	11	1.1	32	10.9
POD 1	154	5.6	110	27	1.4	47	16
POD 2	141	5.3	105	27	1.3	45	15.3
POD 3	133	6	104	27	1.2	42	14.3
POD 4	150	6.1	112	26	1.1	51.9	17.3
POD 5	142	6.2	106	25	1.2	46	15.6
POD 6	131	4.7	99	27	1.1	42	14.3
POD 8	135	5.1	105	24	1.1	41	13.9
POD 10	143	3.2	105	23	1.4	40	13.6

*BUN* blood urea nitrogen, *Cl* chloride, *Cre* creatine, *Hb* hemoglobin, *Hct* hematocrit, *K* potassium, *Na* sodium, *POD* postoperative day.

**Figure 5:**
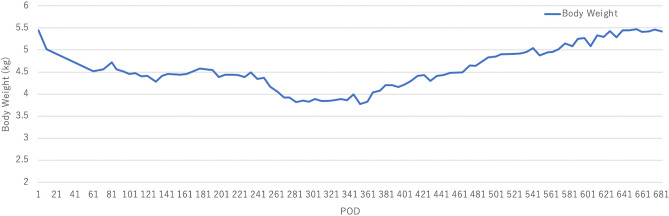
Time course of postoperative body weight in the monkey.

We performed CRH and GRF stimulation tests at 2 postoperative years, which showed cortisol levels at 0.44 μg/dL, with undetectable serum ACTH and GH levels (Table 1). The AVP level was undetectable, even under the high Na level state due to short-term water restriction (Na, 152 mEq/L; AVP, < 0.4 pg/mL) (Table 1). The monkey showed long-term survival without any major complications, including CSF leakage or meningitis. The postoperative course based on body weight is shown in Fig. 5. The body weight decreased slightly after surgery; however, it was stable and the preoperative weight was regained after 2 years with hormone replacement therapy and improved diet. The monkey was euthanatized at 2 postoperative years, and the autopsy showed no remaining pituitary gland.

## Discussion

To reduce the number of monkeys undergoing experimentation, this study evaluated only one case. However, we considered that this case showed a meaningful result for establishing a hypophyseal dysfunction model and the protocol. In prioritizing reduction among the principles of 3Rs (Replacement, Reduction, Refinement) in animal experiments, no additional sacrifice from the monkey was required because we achieved the purpose of this experiment. We also established the protocol for hormone replacement and other adjuvant therapies to achieve long-term survival after hypophysectomy.

### Treatment for anterior pituitary dysfunction

No curative treatment has been established for pituitary dysfunction, which affects the life prognosis ^11^. Impairment of the pituitary gland’s secretion ability could result in a lack of down-stream hormones, such as adrenocortical, thyroid, insulin-like growth factor-1, and gonadal hormones. If left untreated, life-threatening complications, including dehydration, electrolyte abnormality, hypotension, and disturbance of impaired consciousness, can occur. Treating anterior pituitary dysfunction involves a life-long replacement therapy for deficit hormones; therefore, no physiological secretion has been reproduced to date. Regarding ACTH deficiency, demand fluctuates up to ten times, depending on the stress amount and time. When replacement is insufficient, adrenal insufficiency could be life-threatening ^3^. Conversely, when replacement becomes excessive, serious complications, including obesity, hypertension, diabetes mellitus, osteoporosis, susceptibility to infection, and psychoneurotic disease, can occur after several years ^12^. Some prospective studies, using current treatments, showed the adverse effects on life prognosis ^13^. The transplantation of pituitary ACTH cells into humans has not been established.

### Preclinical trial necessity before human application

Previous studies showed transplantation of pituitary ACTH cells differentiating from human embryonic stem (ES) cells. iPSCs can be used to treat hypopituitarism in principle ^3,14^. Particularly, our study showed the therapeutic effect of transplantation using hypophysectomized mice ^3,4^. We transplanted ES-derived pituitary tissue into the kidney’s subcapsular space and evaluated the transplantation’s effect. The pituitary ACTH hormone is secreted from the graft into mouse blood, thus secreting adrenocortical hormone from the mouse adrenal gland in its downstream. The transplanted mice’s spontaneous activity was similarly improved, producing a better life prognosis; however, technological developments and demonstration trials are necessary before application in humans in clinical practice. An important step is a preclinical trial, using medium- or large-sized animals. In rodent transplant experiments, the transplantation site is restricted to the renal capsule or subcutaneous fat layer. Their body size makes transplantation into the sella of the pituitary gland technically impossible. Additionally, pituitary hormones are equally related to cognitive functions, making it difficult to examine their unexpected side effects, necessitating a monkey model.

Furthermore, monkey iPSCs have been established from a cynomolgus monkey of HLA homodonors. Presently, for regenerative medicine, the basic policy involves iPSCs for clinical use being obtained from human HLA homodonors and transplanted into a patient with one fitted HLA side, thereby reducing rejection reaction ^2^, thus using a cynomolgus monkey to create a hypophyseal dysfunction model.

### Surgical methods and complications in creating a hypophyseal dysfunction model in a monkey

Hypophysectomy involves operating via the transcranial, transnasal, or transoral method. The transcranial method requires a large skin incision and brain retraction, potentially causing fatal brain injury. Similarly, we were also concerned about the monkey touching the postoperative wound, resulting in both infection and healing delay. Furthermore, we considered the overall investigation of the pituitary gland difficult without strong brain retraction owing to the sella’s anatomical position.

The pituitary gland’s transnasal approach has been established in humans. However, monkeys generally breathe nasally; therefore, surgically-induced nasal obstruction can impair natural breathing and cause asphyxiation. Moreover, their nasal cavities are too narrow to approach the sella. These anatomical difficulties render the transnasal approach inadequate in monkeys.

The transoral approach does not require retracting important structures, including the brain and cranial nerves. We can achieve a wide surgical corridor by the transoral approach without passing through the narrow nasal cavities; moreover, it seemed possible to reduce the risk of wound contact; therefore, we chose the transoral approach for the removal of the pituitary gland. This approach may cause some complications, such as infection and CSF leakage ^15,16^. There have been a few reports of performing transoral surgery for hypophysectomy in monkeys to date; however, there is no article on its use in cynomolgus monkeys. Tindill et al. performed microscopic transoral surgery in 37 rhesus monkeys ^7^, with high postoperative mortality (only 20 survived). The cause of death was considered as anesthesia in two cases, concluding that the other cause of death was the long operative time or excessive manipulation of the infundibulum of the pituitary gland. We considered that a long operating time and excessive manipulation resulted from the difficulty in determining the surgical route to the pituitary gland under microscopic guidance. We used the navigation system and an endoscopic operation. As a result of the examination by preoperative CT, we similarly found it difficult to intraoperatively identify the monkey’s pituitary gland because the sphenoidal sinus did not contain air, and the sella was extremely small in size. We utilized the navigation system to compensate for these anatomical disadvantages. We considered that minimizing the bone fenestration by measuring the direct route to the sella with the navigation system could reduce surgical invasiveness and postoperative CSF leakage risk. We could observe the operative structure more closely and in detail through an endoscope, reducing residual pituitary tissue and possibility of complications, such as cavernous sinus bleeding, internal carotid artery injury, and hypothalamus damage. Hence, we considered the hypophyseal dysfunction model in cynomolgus monkeys safer and feasible.

### Evaluating pituitary function and the hormone replacement method

Evaluation methods of pituitary function and hormone replacement therapies for hypopituitarism have already been well clinically established in humans. Hence, we evaluated whether these human tests could be applicable to monkeys. We obtained a sufficient response to the hormonal stimulation test. Subsequently, we applied the protocol of human hormone replacement treatment to our cynomolgus monkey model. We considered that an accurate amount of steroid replacement is extremely important for the long-term survival of monkeys with hypopituitarism. The intramuscular administration of steroid hormone was considered more accurate than food administration. Although the administration route differed from humans, the drug dose was the same. With these protocols, our monkey showed complication-free long-term survival. Regarding posterior pituitary function, we could not accurately measure water intake and urine volumes because the monkey played with the bottled water. It probably had DI due to the undetectable AVP levels and hypernatremia resulting from short-term water deprivation required for several evaluations. Additionally, we applied the protocol of human hormone replacement for DI to our cynomolgus monkey model. Our surgical technique did not harm the hypothalamus or disturb the monkey’s thirst and consciousness; therefore, this model could be easily managed by providing the monkey with free access to water and an injection of desmopressin. Our monkey with DI experienced no complication concerning water balance in this study.

### Future prospects

In the future, we will conduct preclinical studies of pituitary cell transplantation using this model and confirm the safety and feasibility of this model.

The developed pluripotent stem cell-derived pituitary cells do not simply secrete hormones, such as ACTH^3,14^. In the cells, ACTH secretion increases in response to superior stimulation such as CRH, while ACTH secretion is suppressed in the sufficient presence of hormones, such as glucocorticoids, at the lower level. Hence, the cells can show feedback mechanism. We also developed cells that secrete GH hormone, which can differentiate the pituitary cells that reproduce the ability to respond to the surrounding environment, and it is presumed that the function of the pituitary gland can be restored by transplanting this into a model such as that of hypophyseal dysfunction. In our differentiation method, ACTH, GH, and various other anterior pituitary cells are included in the differentiated organoids. Therefore, we can evaluate each pituitary hormone axis individually.

In conclusion, we succeeded in creating a hypophyseal dysfunction model in cynomolgus monkey. Endoscopic transoral transsphenoidal surgery with navigation is a minimally invasive and safe surgical method to establish such a model. Long-term survival was achieved with the surgical and hormone replacement methods used in this study; hence, the same protocol can be employed in the future.

## Data Availability

All data generated or analyzed during this study are included in this published article or in the data repositories listed in References.
